# Cost-effectiveness of first-line versus second-line use of brigatinib followed by lorlatinib in patients with ALK-positive non-small cell lung cancer

**DOI:** 10.3389/fpubh.2024.1213318

**Published:** 2024-02-15

**Authors:** Wenjie Liu, Gengwei Huo, Peng Chen

**Affiliations:** ^1^Department of Thoracic Oncology, Tianjin Medical University Cancer Institute and Hospital, Tianjin, China; ^2^National Clinical Research Center for Cancer, Tianjin, China; ^3^Key Laboratory of Cancer Prevention and Therapy of Tianjin, Tianjin, China; ^4^Tianjin’s Clinical Research Center for Cancer, Tianjin, China

**Keywords:** cost-effectiveness analysis, brigatinib, lorlatinib, ALK, non-small cell lung cancer

## Abstract

**Background:**

The ALTA-1 L trial and EXP-3B arm of NCT01970865 trial found that both brigatinib and lorlatinib showed durable and robust responses in treating ALK-positive non-small cell lung cancer (NSCLC) patients. However, brigatinib and lorlatinib treatments are costly and need indefinite administration until the disease progression. Thus, it remains uncertain whether using brigatinib followed by lorlatinib before chemotherapy is cost-effective compared to reserving these two drugs until progression after chemotherapy.

**Methods:**

We used a Markov model to assess clinical outcomes and healthcare costs of treating ALK-positive NSCLC individuals with brigatinib followed by lorlatinib before chemotherapy versus a strategy of reserving these drugs until progression after chemotherapy. Transition probabilities were estimated using parametric survival modeling based on multiple clinical trials. The drug acquisition costs, adverse events costs, administration costs were extracted from published studies before and publicly available data. We calculated lifetime direct healthcare costs, quality-adjusted life-years (QALYs), and incremental cost-effectiveness ratios from the perspective of a United States payer.

**Results:**

Our base-case analysis indicated that the incremental cost-effectiveness ratios of using first-line brigatinib followed by lorlatinib compared with second-line brigatinib followed by lorlatinib is $-400,722.09/QALY which meant that second-line brigatinib followed by lorlatinib had less costs and better outcomes. Univariate sensitivity analysis indicated the results were most sensitive to the cost of brigatinib. Probability sensitivity analysis revealed that using brigatinib followed by lorlatinib before chemotherapy had a 0% probability of cost-effectiveness versus delaying these two drugs until progression after chemotherapy at a willingness-to-pay threshold of $150,000 per QALY. Sensitivity analyses conducted revealed the robustness of this result, as incremental cost-effectiveness ratios never exceeded the willingness-to-pay threshold.

**Conclusion:**

Using brigatinib as first-line treatment followed by lorlatinib for ALK-positive NSCLC may not be cost-effective given current pricing from the perspective of a United States payer. Delaying brigatinib followed by lorlatinib until subsequent lines of treatment may be a reasonable strategy that could limit healthcare costs without affecting clinical outcomes. More mature data are needed to better estimate cost-effectiveness in this setting.

## Introduction

Lung cancer is a prevalent type of malignant tumor globally and is responsible for almost a fifth of all cancer related mortality (1). Non-small cell lung cancer (NSCLC) comprises about 85% of all cases of lung cancer (2). Worse, over 70% of patients are diagnosed with advanced stage NSCLC, resulting in a low 5-year survival rate of only 18% (3, 4). In about 3–5% of NSCLC individuals, the oncogenic anaplastic lymphoma kinase (ALK) gene rearrangement occurs (5–7), which represents a molecularly and clinically diverse subtype of NSCLC sensitive to ALK tyrosine kinase inhibitors (5, 8, 9). Currently, first-line treatment, named patients who have not been exposed to any prior anti-cancer agents, options for ALK-positive NSCLC include crizotinib (10), alectinib (11), or ceritinib (12). Nevertheless, crizotinib’s efficacy is often limited by the development of drug resistance in patients, which can result from secondary mutations in the ALK kinase domain or other ALK independent mechanisms (13). More effective new-generation ALK tyrosine kinase inhibitors have been created to combat crizotinib resistance, and have shown efficacy in the treatment of both newly diagnosed patients and those with ALK-positive NSCLC who have become refractory to crizotinib (11, 12, 14, 15). Despite receiving second-generation tyrosine kinase inhibitors, many patients ultimately develop resistance or exhibit disease progression in the central nervous system (16, 17).

Brigatinib, a highly effective inhibitor of ALK, has been shown to target an extensive ALK mutations and c-ros oncogene 1 (ROS1) rearrangements (18–21). It has also shown significant benefits in improving progression-free survival (22, 23). With promising results seen in individuals of advanced ALK-positive NSCLC, brigatinib underwent testing through the phase III trial called ALTA-1 L, which evaluated brigatinib and crizotinib in the first-line setting for patients with ALK-positive NSCLC (24). After a median follow-up of 11 months, the study showed that brigatinib reduced the likelihood of disease progression by 51% versus crizotinib, with, respectively, evaluated 12-month progression-free survival rates of 67 and 43%. Individuals with measurable lesions also had higher rates of intracranial response with brigatinib (78%) compared to crizotinib (29%). Additionally, no new safety issues were identified during the study (24). The approval of brigatinib in the first-line setting has improved the available treatment options for patients (25–27). Meanwhile, lorlatinib is a highly potent and novel third-generation tyrosine kinase inhibitor selective targeting the ALK and ROS1 kinases (17). In the phase II EXP-3B arm of NCT01970865 trial, named ALK-positive patients with disease progression following one previous non-crizotinib ALK tyrosine kinase inhibitor with any number of chemotherapy regimens, lorlatinib demonstrated significant intracranial and overall activity in subsequent therapy for advanced NSCLC individuals (28). Although using first-line brigatinib followed by lorlatinib in the ALTA-1 L and EXP-3B arm of NCT01970865 trials significantly improved progression-free survival, it comes with a high cost.

Previous studies evaluated the economic value of regimen regarding brigatinib for individuals who have ALK-positive NSCLC (29–31). The incremental cost-effectiveness ratio for these studies varies, for example, Cranmer et al. (29) both applied clinical data from the ALTA-1 L and ALEX trials to assess brigatinib from a United States perspective, which demonstrated that brigatinib provided better results than crizotinib, resulting in an increase of 0.97 quality-adjusted life-years (QALYs). The improved efficacy was linked to a longer time on treatment with brigatinib, leading to higher costs ($210,519 more than crizotinib). The base-case incremental cost-effectiveness ratio was $217,607/QALY gained, while brigatinib was related to cost-savings vs. alectinib. Furthermore, the analysis only provide information about the first-line environment.

Although using first-line brigatinib followed by lorlatinib in the ALTA-1 L and EXP-3B arm of NCT01970865 trials significantly improved PFS, these drugs are highly cost. Thus, this study will be the first to evaluate the cost-effectiveness of using brigatinib followed by lorlatinib before chemotherapy compared to reserving these two drugs until progression after chemotherapy for individuals with ALK-positive advanced NSCLC from the perspective of United States payers.

To achieve this goal, we have constructed a model suitable for simulating the process of chronic diseases, namely Markov model, similar to the treatment process for ALK-positive NSCLC. By calculating the direct medical costs, effectiveness and deriving the incremental cost-effectiveness ratios, then comparing it with the willingness-to-pay threshold. After conducting sensitivity analysis to evaluate the stability of the model, we obtain objective cost-effectiveness characteristics of the treatment options. These findings can assist healthcare providers and hospital administrators in making informed decisions when providing care to cancer patients, specifically from the perspective of United States payers.

## Methods

### Model framework

**Figure 1:**
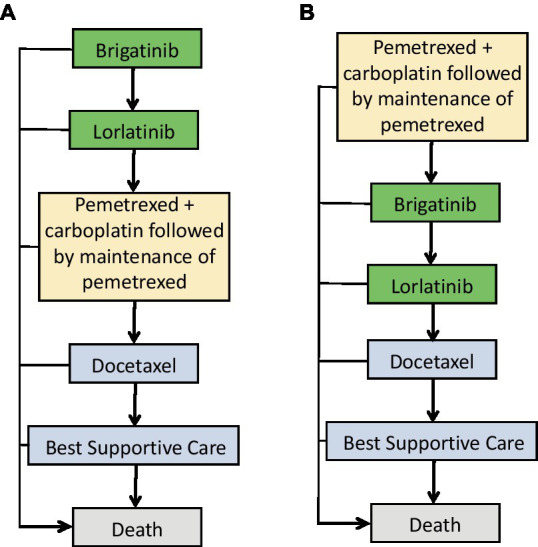
Treatment sequences used in the Markov model. **(A)** Treatment sequence for individuals who receive first-line brigatinib followed by lorlatinib. **(B)** Treatment sequence for individuals who receive second-line brigatinib followed by lorlatinib.

We utilized TreeAge Pro 2022 software (TreeAge, Williamstown, Massachusetts) to implement a Markov model comparing the costs and effectiveness of two treatment strategies for ALK-positive advanced NSCLC: using front-line brigatinib followed by lorlatinib compared with second-line brigatinib followed by lorlatinib. Second-line treatment in oncology refers to the therapeutic approach used after the failure of the initial or first-line treatment for cancer. Mutually exclusive health statuses constituted the model structure, as Figure 1A displays that brigatinib followed by lorlatinib is seen as first-line treatment, while pemetrexed + carboplatin followed by maintenance of pemetrexed is seen as second-line treatment. On the other hand, Figure 1B shows that pemetrexed + carboplatin followed by maintenance of pemetrexed is seen as first-line treatment, while brigatinib followed by lorlatinib is seen as second-line treatment (Figure 1). The transition probabilities in each treatment line were calculated through the Kaplan–Meier curves in progression-free survive, where the probability of death for each treatment line is equal to the natural mortality rate. We modeled over a lifetime horizon with 1-month Markov cycles, estimating costs and utilities related to each therapeutic strategy. Assuming that the corresponding expenditure occurs at the beginning of each cycle, there is no cost adjustment for the half a cycle (32). The incremental cost-effectiveness ratios is calculated using the formula: [Cost (first-line)-Cost (second line)]/[QALY (first-line)-QALY (second line)]. We calculated incremental cost-effectiveness ratios for the main results to represent the expenses in 2023 United States dollar associated with each incremental quality-adjusted life-year (QALY) obtained. Discounting at a rate of 3% annually (33), both utilities and costs were accounted for. The analysis was performed with a willingness-to-pay threshold of $150,000 per QALY from the United States payer’s perspective (34).

### Participants and interventions

Individuals with advanced ALK-positive NSCLC in the ALTA-1 L trial who had not previously been treated with ALK tyrosine kinase inhibitors to receive brigatinib at a daily dose of 180 mg (24) and the EXP-3B arm of NCT01970865 trial (28) involved ALK-positive NSCLC individuals who received one previous non-crizotinib ALK-directed therapy, with or without chemotherapy, to receive lorlatinib at a daily dose of 100 mg. The model followed newly diagnosed individuals with ALK-positive advanced NSCLC who were treated with either brigatinib (24) or cisplatin plus pemetrexed followed by maintenance of pemetrexed (35). In the brigatinib arm, individuals who progressed subsequently received lorlatinib (28), and if they progressed again, they received third-line therapy with cisplatin plus pemetrexed followed by pemetrexed maintenance (35). In the cisplatin plus pemetrexed followed by pemetrexed maintenance arm, individuals who progressed subsequently received brigatinib (24), and if they progressed again, they received third-line treatment with lorlatinib (28). Fourth-line were identical between arms and docetaxel was used (36). Administration schedules and treatment dosing for each therapy line were determined according to respective clinical trials (24, 28, 35, 36). We hypothesized that both arms of patients could receive follow-up therapy until disease progresses. After treatment with docetaxel, patients transitioned into a best supportive care state until their passing.

### Survival model and progression risk estimates

We utilized the GetData Graph Digitizer software package (version 2.22) to extract survival data the progression-free survival Kaplan–Meier curves from each trial. We utilized R software (version 4.2.1) to reconstruct individual patient-level data, and then fitted these reconstructed survival data to various parametric functions such as weibull, exponential, gamma, gompertz, gengamma, log-normal, log-logistic. Based on the Akaike information criterion and Bayesian information criterion, we screened the appropriate parametric distributions for the treatments of brigatinib, lorlatinib, cisplatin plus pemetrexed followed by pemetrexed maintenance, docetaxel, and best supportive care (Supplementary Table 2). Supplementary Figure 1 in the supplement provided information on how well the model fits. We use Microsoft Excel software to calculate time-dependent transition probabilities for the two groups of patient treatment based on data from each trial, and then extrapolated over a lifetime horizon. The transition probabilities values for each model cycle were calculated using the formula: transition probabilities [(*tu*) = 1 – exp. {λ (*t* – *u*)^γ^ – λ*t*^γ^} λ > 0, γ > 0], where “*u*” denotes the model cycle and “tu” denotes the arrival at state “*t*” following “*u*” cycles. To determine the probability of death during each treatment line, we combined an age-matched background mortality rate from United States Life Tables (37) (Supplementary Table 3). Finally, the overall survival data of NSCLC patients who received the best supportive care were used to estimate the transition probabilities of death from the health state of best supportive care (38).

### Cost estimates

We took into account health resource utilization and direct medical expenditure, involving those associated with acquiring and administering medication, managing the disease, and addressing treatment-related adverse events (Supplementary Table 1). The agent dosage was determined using the patient’s body surface area of 1.72 m^2^, weight of 65 kg and creatinine clearance rate of 70 mL/min (39, 40). In the model, only severe adverse events (grade ≥ 3) were considered, such as anemia neutropenia, hypertension, increased blood creatine kinase level and lipase level, and so on (24, 28, 35, 36). Acquisition costs for brigatinib, lorlatinib, cisplatin, pemetrexed, and subsequent therapies were derived from public available databases that are all the most up-to-date in March 2023 (41, 42). We also took into account the expenses of agent administration using the 2022 Centers for Medicare & Medicaid Services Physician Fee Schedule (43). We assumed that patients would undergo regular follow-up, consisting of standard laboratory tests and a physician’s office visit once a month, as well as tumor imaging once every three months (44, 45). The adverse events costs were extracted from published studies before (46). We assumed that if patients experience adverse events, the cost of treating those events would only be recorded for the first cycle, and that the costs for adverse events would be incurred only once. We adjusted inflation all costs using the American consumer price index.

### Utility estimates

QALYs are defined as integrating life span with health-related quality of life, measured as utilities (health state values from 0, indicating death to 1, indicating full health) (47). In the base case, we used a utility of 0.71 for the first-line treatment, 0.67 for second-line therapy, 0.59 for third-line treatment, and 0.46 for fourth-line treatment and best supportive care (48). We used TreeAge Pro 2022 software to calculated QALYs through weighting individuals survival according to utility estimations for every health state. Adverse events leading to disutility values were also considered in this study (49). The overall impact of adverse events on QALYs was applied to the first cycle of the model (50). All utilities parameters are listed in Supplementary Table 1.

### Sensitivity analysis

**Figure 2:**
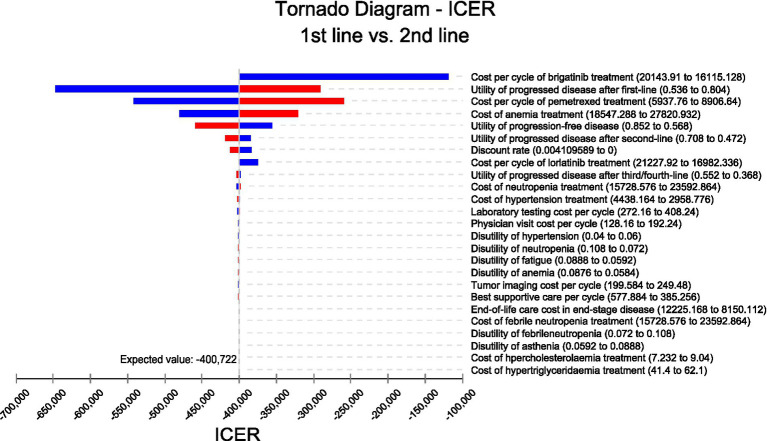
Tornado diagram for univariate sensitivity analyses.

**Figure 3:**
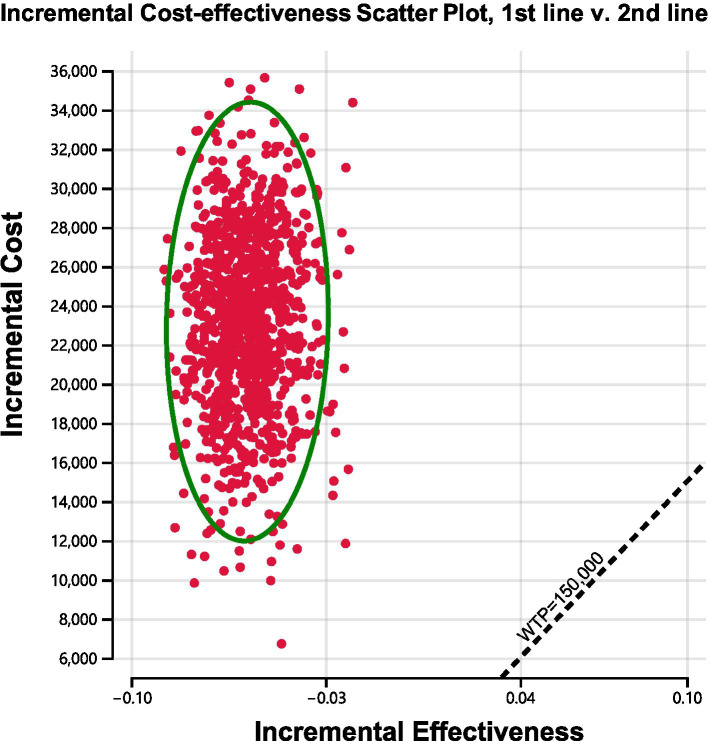
Incremental cost-effectiveness scatter plot diagram for using brigatinib followed by lorlatinib before chemotherapy versus reserving these drugs until after chemotherapy.

**Figure 4:**
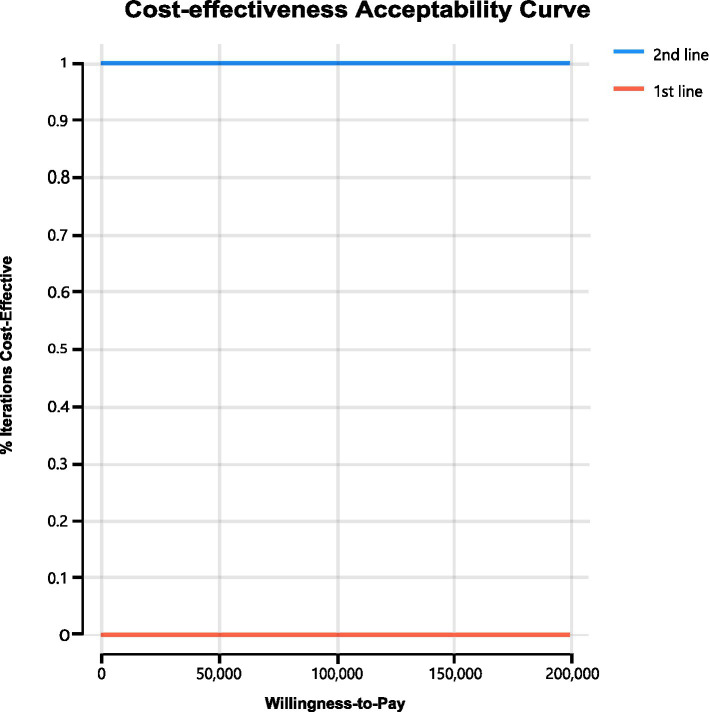
The cost-effectiveness acceptability curves for probabilistic sensitivity analyses.

We conducted sensitivity analyses to investigate how the results were affected by the uncertainty of the parameters. The clinical parameters changed within reasonable limits in the univariate sensitivity analysis, derived from the confidence intervals or assumptions with a 20% variance from the baseline value, as shown in the tornado diagram (Figure 2). We conducted 1,000 Monte Carlo simulations to proceed with the probability sensitivity analysis by random and simultaneous preset parameter variations in accordance with specific distribution patterns (Supplementary Table 1). Due to the real-world performance in the market, it is highly unlikely that the cost of brigatinib or lorlatinib will increase. As a result, only the influence of the price decrease on the incremental cost-effectiveness ratios was analyzed. We used scatter plots and cost-effectiveness acceptability curves to evaluate the cost-effectiveness of each treatment options at various willingness-to-pay thresholds (51, 52) (Figures 3, 4).

## Results

### Base-case analysis

Over a lifetime horizon, the total QALYs for using brigatinib followed by lorlatinib before chemotherapy and reserving these two drugs until progression after chemotherapy were evaluated to be 0.70 and 0.76, respectively. The total costs associated with these treatments were expected to be $219,712 and $196,513, respectively. During that period, not only did the use of brigatinib in the front-line setting followed by lorlatinib not improve QALYs versus reserving brigatinib followed by lorlatinib until later line treatment, but front-line brigatinib followed by lorlatinib was also related to substantially greater lifetime patients healthcare costs, giving rise to an incremental cost-effectiveness ratio of $-400,722.09/QALY (Table 1).

**Table 1:** Base-case results of the model.

Arm	Costs, US $	△Costs, US $	QALYs	△QALYs	ICER US $/QALY
First-line brigatinib followed by lorlatinib	219,712	–	0.70	–	–
Second-line brigatinib followed by lorlatinib	196,513	−23,199	0.76	0.06	−400,722.09

ICER, incremental cost-effectiveness ratio; QALYs, quality-adjusted life-years.

### Sensitivity analysis

As shown in the tornado diagram of ALK-positive advanced NSCLC patients in Figure 2, parameters that most influenced the incremental cost-effectiveness ratio were the cost of brigatinib, cost of pemetrexed, and utility of second-line treatment. Other variables with large or moderate influences were the cost of anemia, utility of first-line treatment, and utility third-line treatment. Despite the variation of various parameters over a wide range, compared to using brigatinib followed by lorlatinib before chemotherapy, the incremental cost-effectiveness ratio of reserving these drugs until after chemotherapy remained less than $150,000/QALY. The robustness of the model results has been confirmed by the fact that there is no overlap between the incremental cost-effectiveness ratio and willingness-to-pay values obtained from varying all parameters within their respective ranges.

As shown in Figure 4, cost-effectiveness acceptability curves indicated that compared with delaying brigatinib followed by lorlatinib until subsequent lines of therapy, the probability of using front-line brigatinib followed by lorlatinib being cost-effective was found to be 0% when the specified willingness-to-pay threshold was $150,000 per QALY gained. As shown in Figure 3, scatter plots described the outcomes of 1,000 simulations of the probabilistic sensitivity analysis, where the majority of outcomes produced fewer QALYs and more costs than second-line brigatinib followed by lorlatinib.

## Discussion

The ALTA-1 L trial and EXP-3B arm of NCT01970865 trial demonstrated that use of brigatinib and lorlatinib in the first line and subsequent line therapy of ALK-positive NSCLC substantially reduced the risk of disease progression (24, 28). Because brigatinib and lorlatinib are high-priced treatments that are administered continuously until the disease progresses, using these treatments in earlier stages of treatment can result in a significant cumulative healthcare expenditure compared to delaying their use until subsequent lines of therapy. Our analysis indicated that prioritizing the use of brigatinib followed by lorlatinib before chemotherapy was not a cost-effective strategy when compared to reserving these drugs until after chemotherapy, given an incremental cost-effectiveness ratio of $-400,722.09 per QALY. From the perspective of United States payers, it was determined through cost-effectiveness acceptability curves (Figure 4) that delaying brigatinib followed by lorlatinib until subsequent lines of therapy was extremely cost-effective for treating the disease. Our analysis of sensitivity indicates that in order for brigatinib to reach widely accepted cost-effectiveness thresholds for cancer therapies and other treatments in the first line setting, a significant reduction in its price would be necessary (33, 34).

One of the most significant factors in the model was the price of brigatinib. When the most sensitive variable changed within a certain range (range, $16115.128–$20143.91 per cycle), compared with using first-line brigatinib followed by lorlatinib, the incremental cost-effectiveness ratios for reserving these two drugs until subsequent line were still lower than $150,000 per QALY, which was considered cost-effective. The utility of second-line treatment was also the most important influencing factor. The utility value adopted in the analysis referred to the published advanced NSCLC patient health utility value data (48). To clarify the impact of health utility value on our model, the ranges of variables were defined for the utility value in the sensitivity analysis (range, 0.536–0.804). The findings illustrated that the upper and lower limits of utility value both make the using first-line brigatinib followed by lorlatinib for individuals with ALK-positive NSCLC not cost-effective. Besides, various parameters such as pemetrexed cost per cycle, cost of anemia treatment or the utility of first-line treatment have no substantial influence on the outcomes of the analysis. The sensitivity analysis focuses on the uncertainty of the model parameters, which confirms the robustness of our model.

It is worth mentioning that, according to our basic model, even if we opt for a more cost-effective approach of deferring the use of brigatinib followed by lorlatinib until later lines treatment, the average direct healthcare costs per patient still end up being excessive. Prior studies have shed light on the significant expenses linked to ALK-positive NSCLC, and this situation is only predicted to exacerbate with the rising adoption of new targeted treatments (53–56). If we acknowledge that our healthcare system cannot bear the cost of each newly developed drug in the market, we need to decide how to ascertain its value. The suggestion is that when formulating policies revolving round national healthcare plans, cost-effectiveness analysis should be employed. Taking into account the unique thresholds of each country, solutions could be explored to ensure that patients receive affordable treatments while pharmaceutical companies are able to make reasonable profits, in order to strike a balance between protecting patients and society from exorbitant financial burdens and supporting the innovation and development of new treatments (57, 58).

Our study in combination with previous researches on cost-effectiveness of cancer treatment (31, 59, 60), emphasizes the urgency to explore alternative pricing strategies due to the high costs associated with cancer agents. Value-based pricing (61), indication-specific pricing (62), or a subscription model (63) could be potential options to explore. There are several regulatory institutes in some countries, including the National Institute for Health and Care Excellence in the United Kingdom, that supervise agents reimbursement and approval according to their established value of economics. However, in the United States, legal regulations mandate that Medicare, the maximum insurer, provide coverage for all approved oncotherapies, limiting negotiations with pharmaceutical companies (64). This has resulted in the cancer agents pricing being not related to their level of innovation or the improvement of overall survival improvement, surrogate endpoints, or quality of life (65). As cancer drug prices continuously rise, updated policy is needed to align costs with proven clinical potency.

This analysis showed uniquely that the use of brigatinib followed by lorlatinib after chemotherapy is superior in terms of cost-effectiveness compared to using brigatinib followed by lorlatinib before chemotherapy. This result emphasizes the significance of taking into account the initiation time of targeted therapy in routine clinical settings, as it could greatly impact its cost-effectiveness. It is necessary to address the issue of which treatment strategy is more cost-effective to use targeted drugs in the primary treatment phase or reserve them for secondary treatment. At present, there is no pharmacoeconomic research that has attempted to answer this question. As far as we know, this study is the first to address this issue. We found that delaying brigatinib followed by lorlatinib until subsequent lines of treatment could curb costs without affecting clinical outcomes, and this finding is particularly significant as it may add more backbone for doctors that are using this way of treatment. In this study, we used Markov models to simulate disease progression as it can account for time-dependent state transitions. In the context of tumor treatment, the disease status of patients changes over time. Markov models can describe the transitions between different states and utilize historical data to estimate transition probabilities. This is essential for evaluating the effectiveness of different treatment strategies and predicting future patient states. Furthermore, Markov models can also consider the impact of different treatment strategies. By considering the patient’s current state and the treatment strategy received, it is possible to predict the patient’s future state and related economic indicators (such as total costs, survival time, etc.). This enables decision-makers to make treatment plan selection and resource allocation decisions based on model results. Despite the clear clinical advantages of using brigatinib as a first-line treatment option, there was no noticeable increase in cost-effectiveness benefits when compared to reserving its use until the treatment of second line. Further researches are required to yield more substantial proof in regards to this matter. The results of our research could prove valuable in the process of negotiating and making decisions regarding national medical insurance. The burden of the current expensive pricing system for anti-cancer drugs in the healthcare insurance system is significant. The high costs of these medications directly contribute to the rising healthcare expenditures. Although both brigatinib and lorlatinib demonstrate notable efficacy, their current prices are excessively high. Considering their cost-effectiveness, a comprehensive evaluation may prove beneficial for healthcare providers and hospital administrators. Based on the findings of this study, healthcare providers can enhance healthcare cost management and explore more cost-effective ways to deliver medical services. They can provide patients with comprehensive information regarding treatment options, including their efficacy, costs, and potential economic benefits of different medication regimens. These results empower patients to make well-informed decisions about their treatment. Hospital administrators can effectively control and reduce these expenses within the framework of the United States healthcare system. Since both first-line and second-line treatments surpass the willingness-to-pay threshold, they can negotiate with suppliers for more reasonable drug prices to decrease treatment costs. Moreover, managers can employ economic evaluation to assess the value of utilizing these drugs in the hospital and identify treatment alternatives that offer relative cost-effectiveness. In light of these findings, informed choices can be made to select treatment plans that not only yield positive outcomes but also align with financial feasibility. This will optimize the quality and efficiency of healthcare services while ensuring the long-term financial sustainability of hospitals.

There are multiple noteworthy advantages to this research. Firstly, we conducted an economic modeling analysis by integrating all the relevant data of clinical trials, which allowed us to compare the cost-effectiveness of using brigatinib followed by lorlatinib before chemotherapy option versus reserving these drugs until after chemotherapy in patients with ALK-positive NSCLC. Secondly, we integrated the treatment paradigms of real world into this model, including using chemotherapy as a subsequent line for individuals with first-line brigatinib followed by lorlatinib failure, the use of subsequent-line brigatinib followed by lorlatinib in patients in whom the first-line chemotherapy had failed, and using docetaxel as a further line for individuals progressing after the third-line therapy.

There were certain limitations in this study that should be acknowledged. Firstly, as most advanced ALK-positive NSCLCs tend to exhibit non-squamous histologic characteristics and there is short of head-to-head comparison data derived from clinical trials, pemetrexed combined cisplatin was screened as the standard chemotherapy regimen for the analysis. The effectiveness of first-line chemotherapy based on pemetrexed has been extensively recorded in cases of NSCLC with ALK positive (66, 67) and the favorable safety profile of cisplatin plus pemetrexed followed by pemetrexed maintenance, which make it an acceptable choice as a control strategy for standard chemotherapy (35). These findings support the selection. However, further cost-effectiveness analyses are encouraged to be performed once head-to-head comparison data from clinical trials become available. Secondly, there is a lack of research on the effectiveness and safety of brigatinib compared to chemotherapy agents. Although indirect comparisons indicated that brigatinib could potentially offer a better balance between efficacy and toxicity, it will be necessary to conduct direct comparative trials between brigatinib and chemotherapy to provide valuable information for the treatment decision making. Thirdly, the study utilized data derived from multiple clinical trials, each with a marginally distinct patient cohort, to assess a lifelong horizon. While we confirmed the model regarding progression-free survival for each therapy line, it was not feasible to verify the predicted survival curves externally due to inadequate long-term survival data available for patients who received brigatinib in the early stages of treatment. It will be critical to evaluate how well our modeled results align with real-world data and long-term clinical trials as such information becomes available and emerges (68). Fourthly, the ALTA-1 L trial and EXP-3B arm of NCT01970865 trial had a varied pool of patients, with variations in mortality risk on an individual patient basis. Fifthly, as there was insufficient quality-of-life data available for brigatinib, we relied on health utility values previously reported in literature for United States NSCLC patients to inform our model. The sensitivity analysis demonstrated that the uncertainty in health utility values did not significantly alter the outcomes. Sixthly, the effectiveness of lorlatinib in subsequent-line setting was obtained from a single arm, non-randomized, phase II trial (EXP-3B arm of NCT01970865 trial) (28). Consequently, our model relies heavily on the validity of the trial, and any potential biases present in the trial will be mirrored in our model. Finally, as the findings of this research were based on a secondary analysis of previously published data, its conclusions may be limited. Addressing the limitations highlighted in this study can lead to significant advancements in the field of ALK-positive NSCLC treatment. Conducting head-to-head comparison trials, cost-effectiveness analyses, and gathering real-world data will help guide treatment decisions and optimize patient outcomes. Additionally, focusing on individual patient risk assessment, collecting quality-of-life data, and implementing robust trial designs can further enhance the validity and applicability of research findings. By incorporating these suggestions into future studies, we can improve the understanding and management of ALK-positive NSCLC, ultimately leading to improved patient care and outcomes.

In conclusion, our study indicated that for patients with ALK-positive NSCLC, a more cost-effective strategy would be to reserve brigatinib followed by lorlatinib until subsequent lines of therapy instead of the use of first-line brigatinib followed by lorlatinib. Substantial price declines or establishing the effectiveness of a fix treatment duration may be required to make brigatinib become a cost-effective in the first-line setting. Mature data for patients receiving brigatinib as a first-line treatment followed by lorlatinib are essential to more fully assess cost-effectiveness in this setting. Our study could provide valuable insights to healthcare policymakers about the optimized treatment options using brigatinib and lorlatinib.

## Data availability statement

The original contributions presented in the study are included in the article/supplementary material, further inquiries can be directed to the corresponding author.

## Author contributions

PC and GH: conception and design. WL and GH: collection, assembly of data, data analysis, interpretation, and manuscript writing. All authors contributed to the article and approved the submitted version.
