# Supplementary material for: Cost-effectiveness of first-line versus second-line use of brigatinib followed by lorlatinib in patients with ALK-positive non-small cell lung cancer
Source: Front Public Health. 2024 Feb 15;12:1213318. doi: 10.3389/fpubh.2024.1213318 (PMC10906082; doi:10.3389/fpubh.2024.1213318)
Supplement: Supplementary file 1 [file Data_Sheet_1.PDF]

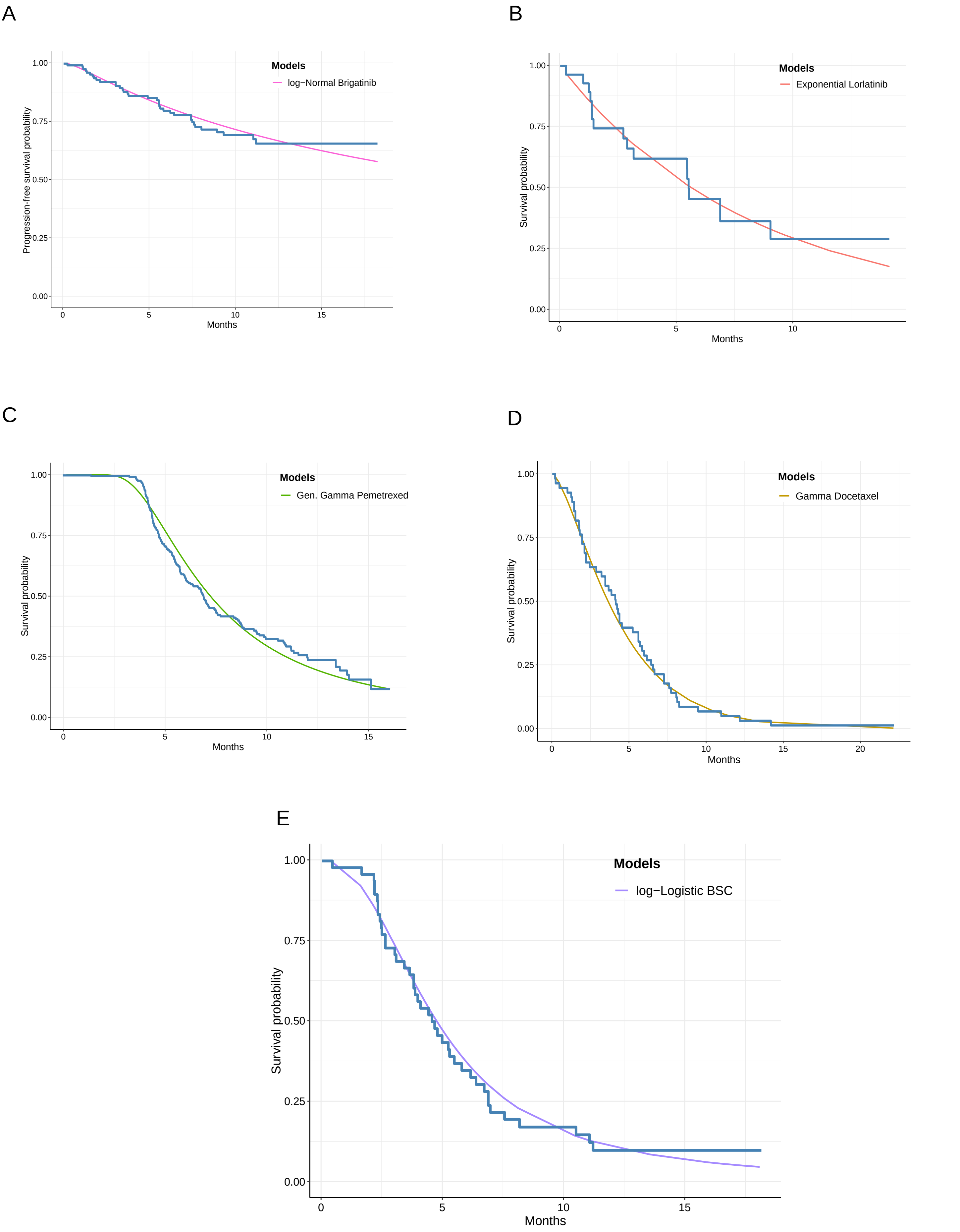

eFigure 1 Parametric distributions for the treatment of (A) brigatinib, (B) lorlatinib, (C) pemetrexed, (D) docetaxel, (E) best supportive care.
