# Supplementary material for: Cost-effectiveness of first-line versus second-line use of brigatinib followed by lorlatinib in patients with ALK-positive non-small cell lung cancer
Source: Front Public Health. 2024 Feb 15;12:1213318. doi: 10.3389/fpubh.2024.1213318 (PMC10906082; doi:10.3389/fpubh.2024.1213318)
Supplement: Supplementary file 2 [file Data_Sheet_2.docx]

**eTable 1 Model parameters and distributions**

| Variable | Baseline value (Reference) | Range | | Distribution |  |
| --- | --- | --- | --- | --- | --- |
|  |  | Minimum | Maximum |  |  |
| Lognormal PFS survival model with brigatinib | Meanlog =3.216; sdlog =1.594 | - | - | - |  |
| Exponential PFS survival model with lorlatinib | rate =0.1198 | - | - | - |  |
| Gengamma PFS survival model with pemetrexed + carboplatin followed by maintenance of pemetrexed | mu =1.825; sigma =0.500; Q=-0.853 | - | - | - |  |
| Gamma PFS survival model with docetaxel | Shape =1.597; rate =0.350 | - | - | - |  |
| Loglogistic PFS survival model with BSC | Shape =2.258; rate =4.731 | - | - | - |  |
| Grade ≥3 AEs incidence in brigatinib therapy |  |  |  |  |  |
| Increased blood creatine kinase level | 0.16 (1) | 0.128 | 0.192 | Beta |  |
| Increased lipase level | 0.13 (1) | 0.104 | 0.156 | Beta |  |
| Hypertension | 0.10 (1) | 0.08 | 0.12 | Beta |  |
| Increased amylase level | 0.05 (1) | 0.04 | 0.06 | Beta |  |
| Grade ≥3 AEs incidence in lorlatinib therapy |  |  |  |  |  |
| Hypercholesterolaemia | 0.15 (2) | 0.12 | 0.18 | Beta |  |
| Hypertriglyceridaemia | 0.16 (2) | 0.128 | 0.192 | Beta |  |
| Grade ≥3 AEs incidence in pemetrexed+cisplatin chemotherapy |  |  |  |  |  |
| Anemia | 0.04 (3) | 0.032 | 0.048 | Beta |  |
| Neutropenia | 0.04 (3) | 0.032 | 0.048 | Beta |  |
| Fatigue | 0.04 (3) | 0.032 | 0.048 | Beta |  |
| Grade ≥3 AEs incidence in docetaxel chemotherapy |  |  |  |  |  |
| Neutropenia | 0.455 (4) | 0.364 | 0.546 | Beta |  |
| Febrile neutropenia | 0.073 (4) | 0.0584 | 0.0876 | Beta |  |
| Anemia | 0.073 (4) | 0.0584 | 0.0876 | Beta |  |
| Asthenia | 0.055 (4) | 0.044 | 0.066 | Beta |  |
| Utility |  |  |  |  |  |
| Progression-free disease | 0.71 (5) | 0.568 | 0.852 | Beta |  |
| Progressed disease after first-line | 0.67 (5) | 0.536 | 0.804 | Beta |  |
| Progressed disease after second-line | 0.59 (5) | 0.472 | 0.708 | Beta |  |
| Progressed disease after third/fourth-line | 0.46 (5) | 0.368 | 0.552 | Beta |  |
| AEs disutility |  |  |  |  | |
| Anemia | 0.073 (6) | 0.0584 | 0.0876 | Beta |  |
| Neutropenia | 0.09 (7) | 0.072 | 0.108 | Beta |  |
| Hypertension | 0.05 (8) | 0.04 | 0.06 | Beta |  |
| Febrile neutropenia | 0.09 (7) | 0.072 | 0.108 | Beta |  |
| Fatigue | 0.074 (7) | 0.0592 | 0.0888 | Beta |  |
| Asthenia | 0.074 (7) | 0.0592 | 0.0888 | Beta |  |
| Drug cost, US$ |  |  |  |  | |
| Brigatinib/cycle | 20143.91 (9) | 16115.128 | 20143.91 | Fixed in PSA |  |
| Lorlatinib/cycle | 21227.92 (9) | 16982.336 | 21227.92 | Fixed in PSA |  |
| Pemetrexed/cycle | 7422.2 (10) | 5937.76 | 8906.64 | Gamma |  |
| Cisplatin/cycle | 23.09 (10) | 18.472 | 27.708 | Gamma |  |
| Docetaxel/cycle | 78.76 (10) | 63.008 | 94.512 | Gamma |  |
| AEs cost, US$ |  |  |  |  | |
| Anemia | 23,184.11 (11) | 18547.288 | 27820.932 | Gamma |  |
| Neutropenia | 19,660.72 (11) | 15728.576 | 23592.864 | Gamma |  |
| Hypertension | 3,698.47 (11) | 2958.776 | 4438.164 | Gamma |  |
| Febrile neutropenia | 19,660.72 (11) | 15728.576 | 23592.864 | Gamma |  |
| Hypercholesterolaemia | 9.04 (12) | 7.232 | 10.848 | Gamma |  |
| Hypertriglyceridaemia | 51.75 (12) | 41.4 | 62.1 | Gamma |  |
| Administration cost per cycle | 155.09 (13) | 124.072 | 186.108 | Gamma |  |
| Tumor imaging cost per cycle | 249.48 (14) | 199.584 | 299.376 | Gamma |  |
| Laboratory testing cost per cycle | 340.20 (14) | 272.16 | 408.24 | Gamma |  |
| End-of-life care cost in end-stage disease | 10,187.64 (15) | 8150.112 | 12225.168 | Gamma |  |
| Physician visit cost per cycle | 160.20 (15) | 128.16 | 192.24 | Gamma |  |
| Best supportive care per cycle | 481.57 (15) | 385.256 | 577.884 | Gamma |  |
| Patients’ weight, kg | 70 (14) | 56 | 84 | Normal |  |
| Patients’ body surface area, m^2^ | 1.82 (13) | 1.456 | 2.184 | Normal |  |
| Creatinine clearance rate (ml/min) | 70 (16) | 56 | 84 | Normal |  |
| Discount rate (%) | 3 (13) | 0 | 5 | Fixed in PSA |  |

**eTable 2 AIC and BIC statistics for alternate parametric survival distributions**

| Distribution | Brigatinib | | Lorlatinib | | Pemetrexed | | Docetaxel | | BSC | |
| --- | --- | --- | --- | --- | --- | --- | --- | --- | --- | --- |
|  | AIC | BIC | AIC | BIC | AIC | BIC | AIC | BIC | AIC | BIC |
| Exponential | 320.515 | 323.435 | **108.144** | **109.477** | 1291.153 | 1295.036 | 278.861 | 280.868 | 247.303 | 249.195 |
| Gamma | 322.248 | 328.088 | 110.043 | 112.707 | 1117.367 | 1125.133 | **274.498** | **278.512** | 241.248 | 245.032 |
| Gengamma | 321.921 | 330.681 | 110.943 | 114.940 | **1072.968** | **1084.618** | 276.470 | 282.492 | 236.871 | 242.546 |
| Gompertz | 322.170 | 328.010 | 110.010 | 112.675 | 1213.91 | 1221.677 | 279.067 | 283.082 | 249.264 | 253.048 |
| Weibull | 322.376 | 328.216 | 110.115 | 112.779 | 1150.005 | 1157.772 | 275.203 | 279.217 | 244.607 | 248.391 |
| Log-logistic | 321.198 | 327.038 | 109.237 | 111.901 | 1104.441 | 1112.208 | 278.283 | 282.298 | **232.492** | **236.275** |
| Log-normal | **319.991** | **325.831** | 108.943 | 111.608 | 1092.503 | 1100.269 | 281.078 | 285.093 | 235.079 | 238.863 |

**eTable 3 Background mortality rate**

| **Age** | **Background mortality rate** | **Age** | **Background mortality rate** | **Age** | **Background mortality rate** |
| --- | --- | --- | --- | --- | --- |
| 26 | 0.000968 | 51 | 0.004484 | 76 | 0.03287 |
| 27 | 0.000994 | 52 | 0.004874 | 77 | 0.036315 |
| 28 | 0.001024 | 53 | 0.005302 | 78 | 0.040253 |
| 29 | 0.001058 | 54 | 0.005771 | 79 | 0.044908 |
| 30 | 0.001095 | 55 | 0.006274 | 80 | 0.049974 |
| 31 | 0.001132 | 56 | 0.006793 | 81 | 0.055475 |
| 32 | 0.001171 | 57 | 0.007321 | 82 | 0.061509 |
| 33 | 0.001213 | 58 | 0.007854 | 83 | 0.068675 |
| 34 | 0.00126 | 59 | 0.008403 | 84 | 0.076701 |
| 35 | 0.001319 | 60 | 0.008999 | 85 | 0.085469 |
| 36 | 0.001389 | 61 | 0.009652 | 86 | 0.095935 |
| 37 | 0.001467 | 62 | 0.010341 | 87 | 0.107533 |
| 38 | 0.00155 | 63 | 0.011056 | 88 | 0.120347 |
| 39 | 0.001639 | 64 | 0.011804 | 89 | 0.134457 |
| 40 | 0.001743 | 65 | 0.012598 | 90 | 0.149939 |
| 41 | 0.001864 | 66 | 0.013484 | 91 | 0.166861 |
| 42 | 0.002001 | 67 | 0.014501 | 92 | 0.185276 |
| 43 | 0.002159 | 68 | 0.015701 | 93 | 0.205223 |
| 44 | 0.002345 | 69 | 0.017146 | 94 | 0.226719 |
| 45 | 0.002547 | 70 | 0.018855 | 95 | 0.24976 |
| 46 | 0.002778 | 71 | 0.020762 | 96 | 0.274312 |
| 47 | 0.003059 | 72 | 0.022816 | 97 | 0.300311 |
| 48 | 0.003391 | 73 | 0.02501 | 98 | 0.327661 |
| 49 | 0.003753 | 74 | 0.027353 | 99 | 0.356235 |
| 50 | 0.004118 | 75 | 0.029897 | 100+ | 1 |

**References**

1. Camidge DR, Kim HR, Ahn MJ, Yang JC, Han JY, Lee JS, et al. Brigatinib versus Crizotinib in ALK-Positive Non-Small-Cell Lung Cancer. *N Engl J Med*. (2018) 379:2027-39. doi: 10.1056/NEJMoa1810171

2. Solomon BJ, Besse B, Bauer TM, Felip E, Soo RA, Camidge DR, et al. Lorlatinib in patients with ALK-positive non-small-cell lung cancer: results from a global phase 2 study. *Lancet Oncol*. (2018) 19:1654-67. doi: 10.1016/S1470-2045(18)30649-1

3. Paz-Ares L, de Marinis F, Dediu M, Thomas M, Pujol JL, Bidoli P, et al. Maintenance therapy with pemetrexed plus best supportive care versus placebo plus best supportive care after induction therapy with pemetrexed plus cisplatin for advanced non-squamous non-small-cell lung cancer (PARAMOUNT): a double-blind, phase 3, randomised controlled trial. *Lancet Oncol*. (2012) 13:247-55. doi: 10.1016/S1470-2045(12)70063-3

4. Cortot AB, Audigier-Valette C, Molinier O, Le Moulec S, Barlesi F, Zalcman G, et al. Weekly paclitaxel plus bevacizumab versus docetaxel as second- or third-line treatment in advanced non-squamous non-small-cell lung cancer: Results of the IFCT-1103 ULTIMATE study. *Eur J Cancer*. (2020) 131:27-36. doi: 10.1016/j.ejca.2020.02.022

5. Chouaid C, Agulnik J, Goker E, Herder GJ, Lester JF, Vansteenkiste J, et al. Health-related quality of life and utility in patients with advanced non-small-cell lung cancer: a prospective cross-sectional patient survey in a real-world setting. *J Thorac Oncol*. (2013) 8:997-1003. doi: 10.1097/JTO.0b013e318299243b

6. Westwood M, Joore M, Whiting P, van Asselt T, Ramaekers B, Armstrong N, et al. Epidermal growth factor receptor tyrosine kinase (EGFR-TK) mutation testing in adults with locally advanced or metastatic non-small cell lung cancer: a systematic review and cost-effectiveness analysis. *Health Technol Assess*. (2014) 18:1-166. doi: 10.3310/hta18320

7. Nafees B, Stafford M, Gavriel S, Bhalla S, Watkins J. Health state utilities for non small cell lung cancer. *Health Qual Life Outcomes*. (2008) 6:84. doi: 10.1186/1477-7525-6-84

8. Nafees B, Lloyd AJ, Dewilde S, Rajan N, Lorenzo M. Health state utilities in non-small cell lung cancer: An international study. *Asia Pac J Clin Oncol*. (2017) 13:e195-195e203. doi: 10.1111/ajco.12477

9. The Drugs.com Drug Information Database. Available at: https://www.drugs.com/price-guide/ (Accessed March 2023) .

10. CMS. The Centers for Medicare & Medicaid Services. Available at: https://www.cms.gov/ (Accessed March 2023) .

11. Wan X, Luo X, Tan C, Zeng X, Zhang Y, Peng L. First-line atezolizumab in addition to bevacizumab plus chemotherapy for metastatic, nonsquamous non-small cell lung cancer: A United States-based cost-effectiveness analysis. *Cancer*. (2019) 125:3526-34. doi: 10.1002/cncr.32368

12. Li S, Li J, Peng L, Li Y, Wan X. Cost-Effectiveness of Lorlatinib as a First-Line Therapy for Untreated Advanced Anaplastic Lymphoma Kinase-Positive Non-Small Cell Lung Cancer. *Front Oncol*. (2021) 11:684073. doi: 10.3389/fonc.2021.684073

13. Lin S, Luo S, Zhong L, Lai S, Zeng D, Rao X, et al. Cost-effectiveness of atezolizumab plus chemotherapy for advanced non-small-cell lung cancer. *Int J Clin Pharm*. (2020) 42:1175-83. doi: 10.1007/s11096-020-01076-3

14. Ding D, Hu H, Li S, Zhu Y, Shi Y, Liao M, et al. Cost-Effectiveness Analysis of Durvalumab Plus Chemotherapy in the First-Line Treatment of Extensive-Stage Small Cell Lung Cancer. *J Natl Compr Canc Netw*. (2021) 19:1141-7. doi: 10.6004/jnccn.2020.7796

15. Liu Q, Luo X, Yi L, Zeng X, Tan C. First-Line Chemo-Immunotherapy for Extensive-Stage Small-Cell Lung Cancer: A United States-Based Cost-Effectiveness Analysis. *Front Oncol*. (2021) 11:699781. doi: 10.3389/fonc.2021.699781

16. Criss SD, Mooradian MJ, Watson TR, Gainor JF, Reynolds KL, Kong CY. Cost-effectiveness of Atezolizumab Combination Therapy for First-Line Treatment of Metastatic Nonsquamous Non-Small Cell Lung Cancer in the United States. *JAMA Netw Open*. (2019) 2:e1911952. doi: 10.1001/jamanetworkopen.2019.11952
